# Genetic relatedness of ceftriaxone-resistant and high-level azithromycin resistant *Neisseria gonorrhoeae* cases, United Kingdom and Australia, February to April 2018

**DOI:** 10.2807/1560-7917.ES.2019.24.8.1900118

**Published:** 2019-02-21

**Authors:** Amy V Jennison, David Whiley, Monica M Lahra, Rikki M Graham, Michelle J Cole, Gwenda Hughes, Helen Fifer, Monique Andersson, Anne Edwards, David Eyre

**Affiliations:** 1 Forensic and Scientific Services, Queensland Department of Health, Brisbane, Queensland, Australia; 2 The University of Queensland, Brisbane, Queensland, Australia; 3 Pathology Queensland Central Laboratory, Brisbane, Australia; 4 New South Wales Health Pathology, Microbiology Randwick, The Prince of Wales Hospital, New South Wales, Australia; 5 The University of New South Wales, Sydney, Australia; 6 National Infection Service, Public Health England, London, United Kingdom; 7 Institute of Tropical Medicine, University of São Paulo, Brazil; 8 Oxford University Hospitals NHS Foundation Trust, Oxford, United Kingdom; 9 Nuffield Department of Medicine, University of Oxford, Oxford, United Kingdom; 10 Big Data Institute, University of Oxford, Oxford, United Kingdom

**Keywords:** Neisseria gonorrhoeae, microbial drug resistance, whole genome sequencing, PCR, United Kingdom, Australia, XDR, antimicrobial resistance, AMR

## Abstract

Between February and April 2018, three ceftriaxone-resistant and high-level azithromycin-resistant *Neisseria gonorrhoeae* cases were identified; one in the United Kingdom and two in Australia. Whole genome sequencing was used to show that the isolates from these cases belong to a single gonococcal clone, which we name the A2543 clone.

Between February and April 2018, *Neisseria gonorrhoeae* (NG) isolates displaying ceftriaxone resistance and high-level azithromycin resistance were independently reported from both the United Kingdom (UK) (one case) and Australia (two cases) [1,2]. Here, genomic analysis is used to demonstrate that these cases were caused by a single clone of extensively drug-resistant (XDR) NG. This study has demonstrated that a single XDR clone, with epidemiological links to Asia, has been successfully transmitted on least three independent occasions and may be still circulating, which is of global public health significance.

## Case description

Of three XDR NG cases, the first was identified in February 2018, in a heterosexual male in the UK, presenting with urethral discharge, who reported sexual contact with a female resident in Thailand in January 2018 [1]. NG was cultured from both a urethral swab taken at the first clinical presentation (G97687) and a pharyngeal swab taken during follow-up (G7944). The two Australian cases were identified in March and April 2018. One case was a male experiencing urethral discharge, who reported sexual contact with a female in south-east Asia, the other case was female, who presented with cervicitis, with no travel history outside of Australia [2].

## Extensive drug resistance

First line treatment for NG typically involves a dual treatment regime of ceftriaxone (500mg intramuscularly) and azithromycin (1 or 2g orally). While in the past 10 years, there have been numerous reports of sustained transmission of high-level azithromycin resistant NG, there has only been several different gonococcal strains observed exhibiting ceftriaxone resistance and these have been sporadic (i.e. limited or no further reports of transmission). Prior to the three cases reported here, no ceftriaxone resistant isolates have additionally exhibited high-level resistance to azithromycin (minimum inhibitory concentration (MIC) ≥ 256 mg/L) [3-8]. Isolates from the three cases exhibited both resistance to ceftriaxone (0.25–0.5 mg/L) and high-level resistance to azithromycin according to European Committee on Antimicrobial Susceptibility Testing (EUCAST) resistance breakpoints, requiring the patients to undergo extensive follow-up and treatments including days of hospitalisation and intravenous therapies [1,2]. All three patients were subsequently followed up for test of cure and confirmed to have cleared their infection.

## Genomic analysis

The pharyngeal isolate from the UK case has been recently assigned as the World Health Organization (WHO) reference strain Q (WHO-Q; NCTC 14208), while the two isolates from the Australian cases represent a cluster referred to as the A2543 clone [1,2]. Isolates from each of the three cases were independently whole genome sequenced in the UK and Australia (European Nucleotide Archive (ENA) PRJEB26560 for UK urethral (G97687) and pharyngeal (G7944) isolates, and PRJEB29480 for Australian male (A2735) and female (A2543) case isolates). All four isolates shared the same NG-multi-antigen sequence type (NG-MAST) (ST 16848), multilocus sequence type (MLST) (ST 12039) and NG-Sequence Typing for Antimicrobial Resistance type (NG-STAR 996) [5]. The isolates harboured a mosaic *penA* allele, type 60.001, conferring ceftriaxone resistance as well as four copies of the 23S rRNA A2059G mutation responsible for high-level azithromycin resistance [9,10]. These findings suggest the UK and Australian isolates are all closely related, however the exact phylogenetic relationship between them has not yet been determined.

## Phylogenetic investigation

We mapped UK and Australian sequence reads against a hybrid assembly reference generated from one of the UK isolates, G97687, and compared the sequences as described previously [1]. The Figure shows a recombination-corrected maximum likelihood phylogeny of the four sequences from the three XDR NG cases and the six most closely related publicly available sequences in the ENA and National Center for Biotechnology Information (NCBI) short read archives. Sequences from the two isolates from the UK case and the isolate from female Australian case were indistinguishable; there was only one single nucleotide polymorphism (SNP) between these three isolates and the male Australian case.

**Figure:**
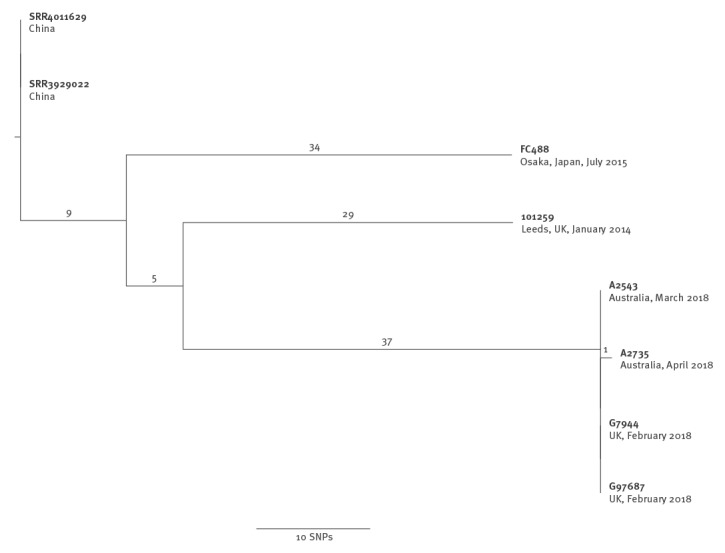
Phylogeny of United Kingdom and Australian sequences obtained in February–April 2018 and the most closely related existing *Neisseria gonorrhoea* genome sequences

SNPs: single nucleotide polymorphisms; UK: United Kingdom.

The figure shows a recombination-corrected maximum likelihood phylogeny (drawn using PhyML [14] and ClonalFrameML [15] as described in [12]) based on mapping to a novel hybrid assembly of one of the UK isolates (G97687). Branch lengths are labelled in SNPs.

The most closely related comparison sequences from the short read archive were all high-level azithromycin resistant, but lacked the *penA* allele seen in the UK and Australian cases [11]. These comparison sequences were > 60 SNPs different, consistent with having a common ancestor several years earlier, based on estimated rates of evolution for NG of around 3.6 SNPs per genome per year [12]. No more closely related sequences are currently available.

## Discussion

The first three ceftriaxone-resistant, high-level azithromycin resistant NG isolates seen worldwide were isolated within a two-month period of each other (February–April 2018). The mechanisms of resistance, including the *penA* 60.001 allele associated with ceftriaxone resistance, and molecular genotypes have been reported elsewhere [1,2], however a detailed phylogenetic analysis between the isolates remained to be performed. In this study, we have demonstrated that isolates from the three cases are tightly genetically clustered and distinct from other globally distributed publicly available NG sequences. The limited SNP differences between the XDR isolates provides compelling evidence that the UK and Australian NG cases are highly related and of the same gonococcal clone. The isolation of these A2543 clone XDR gonococci within a short time period, with epidemiological links to south-east Asia in two of three cases, suggests that this clone may be circulating in Asia, which is concerning. It is important to note that the both the UK and Australia have active NG antimicrobial resistance (AMR) surveillance programs, which were instrumental to the culture, detection and appropriate follow up of these cases. Further, while these cases likely only represent sporadic importations into the UK and Australia, it is highly probable that the clone is present elsewhere, but may not have been detected due to lack of testing and surveillance.

Further spread of this A2543 XDR strain would have serious implications for the current first-line NG treatment recommendations. The multiple detections of the A2543 clone, including asymptomatically in the pharynx in one case highlights the need to improve culture-based NG AMR surveillance in many regions, as well as ensuring clinicians collect pharyngeal samples – a site important from both transmission and treatment clearance perspectives. We have described a PCR that can detect the *penA* 60.001 allele associated with both the A2543 clone and FC428 strains [13] and which could be used for direct molecular screening to further enhance surveillance activities. International collaboration is key to assisting in the prevention and control of what may be an emerging global public health threat.
